# The end-expiratory occlusion test: please, let me hold your breath!

**DOI:** 10.1186/s13054-019-2554-y

**Published:** 2019-08-07

**Authors:** Francesco Gavelli, Jean-Louis Teboul, Xavier Monnet

**Affiliations:** 1 Service de médecine intensive-réanimation, Hôpital de Bicêtre, Hôpitaux Universitaires Paris-Sud, 78, rue du Général Leclerc, F-94270 Le Kremlin-Bicêtre, France; 2 Faculté de médecine Paris-Sud, Université Paris-Sud, Inserm UMR S_999, F-94270 Le Kremlin-Bicêtre, France; 3 Department of Translational Medicine, Emergency Medicine Unit, Università degli Studi del Piemonte Orientale, 28100 Novara, Italy

## Introduction

Fluids must be considered as drugs, with serious adverse effects and inconstant efficacy. Then, they should be administered only if there is reasonable chance that cardiac output (CO) will increase in response. Many tests or indices detecting “fluid responsiveness” have been developed for this purpose.

With some of these tests, the relationship between CO and cardiac preload is assessed through the haemodynamic effects of mechanical ventilation. It is the case for the end-expiratory occlusion (EEO) test, which has already been investigated in a reasonable number of studies [1–13]. In this commentary, we will explore its haemodynamic effects, review the literature validating it and describe its practical modalities.

## What’s behind EEO?

Basically, the test consists in interrupting the ventilator at end-expiration for 15–30 s and assessing the resulting changes in CO. During positive pressure ventilation, insufflation increases the intrathoracic pressure, which is transmitted to the right atrial pressure, the backward pressure of venous return. The right cardiac preload decreases.

As ventilation is stopped in expiration, at the level of positive end-expiratory pressure (PEEP), the cyclic impediment in venous return is interrupted and the right cardiac preload reaches its maximum. If EEO is long enough, the increase in right cardiac preload is transmitted to the left side. An increase in stroke volume and CO in response may indicate preload responsiveness of both ventricles.

## Is the EEO test reliable?

In 2009, our group showed in critically ill patients that an increase in CO ≥ 5% during a 15-s EEO reliably predicted its response to a 500-mL saline infusion [1]. Among 12 further studies, all but two confirmed these results [2–13], with areas under the receiver operating characteristic curve (AUROC) ranging from 0.90 [13] to 1.00 [11]. In two studies, the test was reliable if performed with a tidal volume at 8 and not 6 mL/kg [9, 12]. In the two “negative” studies, performed with tidal volumes of 8.2 mL/kg [5] and 6 mL/kg [7], AUROC were 0.78 and 0.65, respectively. Among all studies, the diagnostic threshold of the EEO-induced increase in CO was 5% on average.

## How to detect EEO-related effects?

The effects of the EEO test must be observed on CO or its surrogates. Arterial pulse pressure, which reflects stroke volume, has been used in one study [1] with good results which, however, need confirmation. A direct measurement of CO is more suitable, but it must be real time and precise. The pulse contour analysis method provides a beat-to-beat estimation of CO and is very precise. It has been used in most of the studies validating the EEO test [1–4, 6–9, 11, 12]. Other techniques that monitor CO non-invasively and continuously, such as the volume-clamp-derived or the plethysmography-derived [14] estimations of CO will probably be also tested.

Ultrasound techniques, oesophageal Doppler and echocardiography, monitor CO beat-to-beat, but are not very precise. The least significant change of the velocity time integral (VTI) obtained using echocardiography is 10% only [15], which might be too large compared to the 5% diagnostic threshold of the EEO test. To overcome this issue, our group has proposed to combine the results of two tests sequentially performed: 15-s EEO and 15-s end-inspiratory occlusion (EIO) [8]. The hypothesis was that EEO should increase VTI in preload responsive patients, whereas EIO should decrease VTI in these patients [8]. We have shown that when the percent changes in VTI induced by EEO and EIO were added, the “EEO+EIO” test was as reliable as the EEO test alone, but with a threshold of 13%, which is more compatible with the precision of the technique. We have reported similar results using oesophageal Doppler [11].

## How should we perform EEO in practice?

**Fig. 1:**
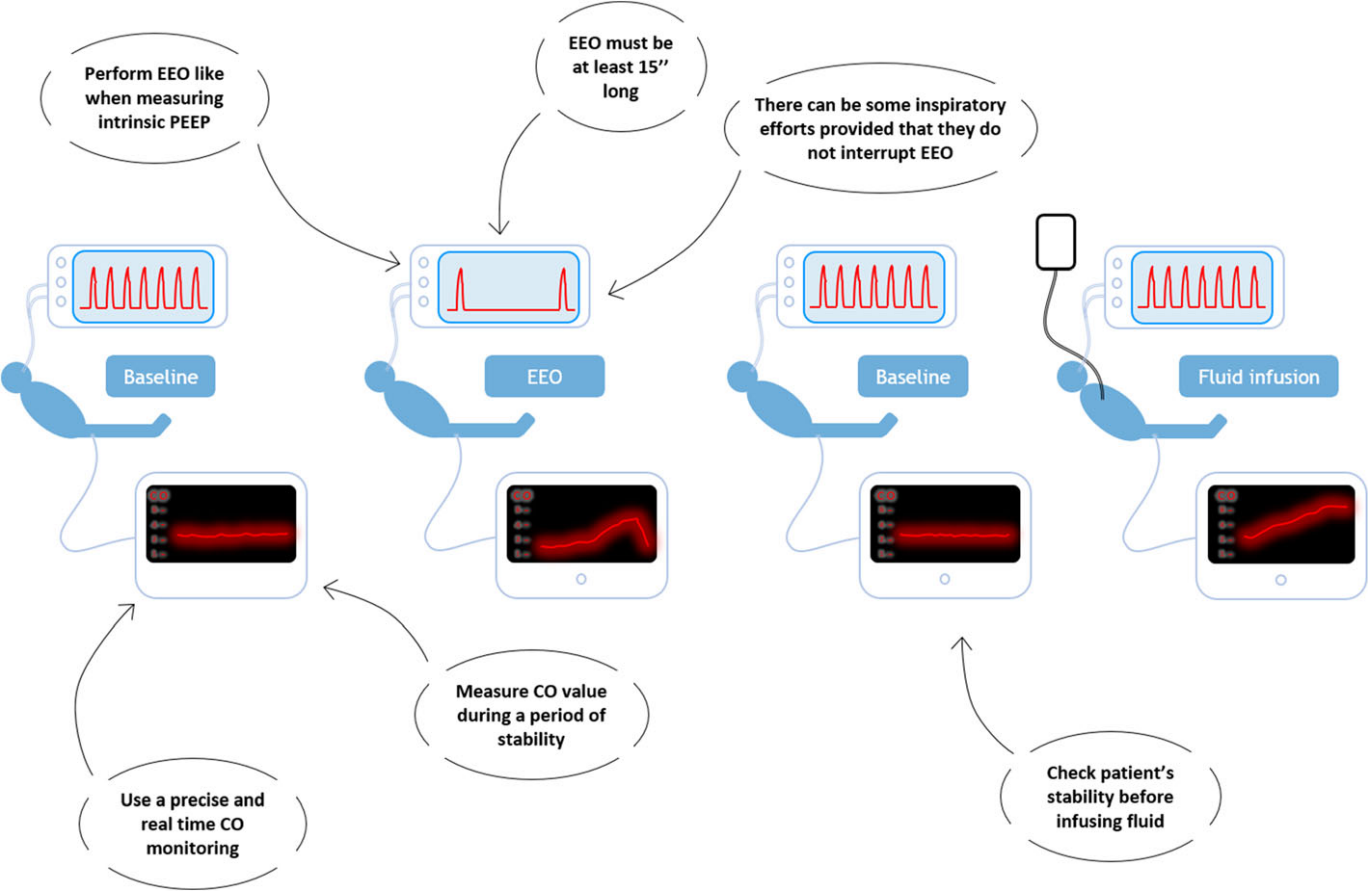
Procedure to perform an end-expiratory occlusion test

After a period of stability, the EEO should be started and maintained for 15 s at least (some studies used either 20-s [13] or 30-s EEO [6, 12]) (Fig. 1). The maximal change in CO appears during the last seconds of a 15-s EEO, and the percentage change compared to the baseline value can be calculated. Finally, after the test, one should check that the CO returns to baseline. One must always keep in mind that there is no test with perfect diagnostic ability. In patients in whom the risk of fluid infusion is particularly high, using another test of fluid responsiveness may ensure the diagnosis.

## Limitations?

### Intense spontaneous breathing activity

A 15-s respiratory hold cannot be sustained by some conscious patients. However, the test can be performed even in some patients who are mildly sedated. Of course, the test is not suitable for patients without mechanical ventilation.

### PEEP level

During EEO, the airway pressure is reduced to PEEP, and the latter may affect EEO reliability. Nevertheless, a study has shown that the reliability was similar for a PEEP at 5 cmH_2_O and at 14 cmH_2_O [4]. Thus, in the range which is used today, the reliability of EEO may not depend on the PEEP level.

### Low tidal volume

Two studies reported that the EEO test was reliable at a tidal volume of 8 but not of 6 mL/kg [9, 12]. Nevertheless, since many studies confirming the reliability of the EEO test included patients with tidal volumes below 8 mL/kg and even less than 7 mL/kg [1–4, 6, 8, 10, 11, 13], this point certainly deserves further investigations.

### Prone position

The only study that investigated the EEO test in prone position found a poor reliability [7]. Sensitivity and specificity were acceptable only in patients in whom central venous pressure increased during EEO. Since there is no obvious reason why the test should be less reliable in prone than in supine position, this result should be confirmed.

## Conclusion

There is growing evidence that the EEO test reliably detects preload responsiveness. It is easier to perform than passive leg raising and has less limitations than pulse pressure variations, provided that a 15-s respiratory hold can be maintained.

## Data Availability

Not applicable.
